# The role of myeloid derived suppressor cells in musculoskeletal disorders

**DOI:** 10.3389/fimmu.2023.1139683

**Published:** 2023-03-03

**Authors:** Yi Ren, Henrik Bäcker, Michael Müller, Arne Kienzle

**Affiliations:** ^1^ Center for Musculoskeletal Surgery, Clinic for Orthopedics, Charité University Hospital, Berlin, Germany; ^2^ Department of Orthopedics, Auckland City Hospital, Auckland, New Zealand; ^3^ BIH Charité Clinician Scientist Program, BIH Biomedical Innovation Academy, Berlin Institute of Health, Charité — Universitätsmedizin Berlin, Berlin, Germany

**Keywords:** myeloid derived suppressor cell (MDSC), bone metabolism, osteoclast, osteoblast, immune cells, inflammation, osteoimmunology

## Abstract

The immune system is closely linked to bone homeostasis and plays a pivotal role in several pathological and inflammatory conditions. Through various pathways it modulates various bone cells and subsequently sustains the physiological bone metabolism. Myeloid-derived suppressor cells (MDSCs) are a group of heterogeneous immature myeloid-derived cells that can exert an immunosuppressive function through a direct cell-to-cell contact, secretion of anti-inflammatory cytokines or specific exosomes. These cells mediate the innate immune response to chronic stress on the skeletal system. In chronic inflammation, MDSCs act as an inner offset to rebalance overactivation of the immune system. Moreover, they have been found to be involved in processes responsible for bone remodeling in different musculoskeletal disorders, autoimmune diseases, infection, and cancer. These cells can not only cause bone erosion by differentiating into osteoclasts, but also alleviate the immune reaction, subsequently leading to long-lastingly impacted bone remodeling. In this review, we discuss the impact of MDSCs on the bone metabolism under several pathological conditions, the involved modulatory pathways as well as potential therapeutic targets in MDSCs to improve bone health.

## 1 Introduction

Bone is a versatile organ that is an essential component for the ambulatory ability and is host to essential cell lineages such as hematopoietic stem cells, as well as bone cells and immune cells. The solid bone matrix is constantly being remodeled in response to changes in physical stress (1). This self-regulated biological remodeling process is mainly driven by bone resorption and formation. While osteoclasts (OCs) eliminate damaged or aged bone tissue, osteoblasts (OBs) are responsible for secretion of new bone matrix and mediation of matrix calcification (2). Both cell types are vital for responding to biomechanical or metabolic changes, remodeling the microstructure of the bone accordingly, and maintaining bone homeostasis.

This equilibrium is governed by several cells and mediating cytokines (3). In particular, the immune system interacts tightly with the bone metabolism (4–6). However, in various pathologies such as tumor metastasis or local inflammation, this delicate equilibrium is distorted (7, 8). Besides focusing on the causative disease, recent research has also focused on identifying key regulatory players to influence bone homeostasis (9, 10). Myeloid derived suppressor cells (MDSCs), a group of immature cells of the myeloid lineage, represent a cell type with immune regulatory function through interaction with effector or regulatory lymphocytes. These cells are activated and proliferate in diseases, including chronic bacterial infection, autoimmune diseases, and cancer (11–15).

Recent studies have described the role of MDSCs in bone-related disease. Bone lesions ranging from systemic bone loss (osteoporosis, autoimmune diseases) to local destruction (osteomyelitis, implant related infection, bone fracture and bone metastasis of tumor) can create a long-lasting inflammatory environment (4, 6, 16, 17). These signals play a key role in myeloid lineage cell activation and differentiation to MDSCs, which in turn impact disease progression and the regenerative capabilities of bone. MDSCs can interact with nearby lymphocytes in the bone, indirectly influencing the bone metabolism through stimulation of the immune system. Additionally, MDSCs were found to impact bone directly, i.e., by differentiating into osteoclasts, or secreting cytokines. In this review, we aim to illustrate how MDSCs can affect bone health and their role in musculoskeletal morbidities.

## 2 Bone remodeling and its interaction with the immune system

Bone serves as one of the most important immune organs as the origin of several immune cells is the bone cavity and its metabolic activity is closely linked to the immune system. The recently coined term “osteoimmunology” connects the metabolic activity of the bone with the immune system (18). The bone forms a relatively closed space that supplies a suitable cradle for the reciprocal interactions of immune cells and bone cells.

Mediators secreted by bone cells can either stimulate or obstruct processes of immune development. Bone cells contribute to the maturation and expansion of various immune cells derived from hematopoietic stem cells (HSCs). Mesenchymal stromal cells expressing the C-X-C motif chemokine-12 (CXCL-12) are required for HSC maintenance (19). Additionally, OBs are essential in maintaining common lymphoid progenitors (CLPs) through expression of IL-7 and CXCL-12 (20). Ablation of OBs results in severely decreased hematopoiesis in the bone marrow, in particular the generation of B cells (21). Osteocytes also support the lymphocyte development and show positive impact on B cell generation (22, 23). Moreover, OCs are fundamental to create bone marrow cavities sufficient in size for HSCs to sustain their physiological capabilities and indirectly support HSCs by recruiting osteoblasts (24). They are also engaged in establishing a livable milieu in the bone to induce HSC homing and niche formation (25).

At the same time the immune system has significant impact on bone homeostasis (26). Over- or under-regulation of the immune system results in abnormal bone mineralization through different mechanisms. Different T cell populations including CD8+, CD4+ T helper cells (Th), and regulatory T cells (Treg) impact the bone metabolism through secretion of various cytokines. CD8+ T cells and Th17 favor osteoclastogenesis by secretion of tumor necrosis factor-α (TNF-α) and IL-17 (17, 27). B cells, as supportive regulators of osteoclasts, limit bone remodeling (28, 29). Macrophages are characterized into two phenotypes, proinflammatory M1 and anti-inflammatory M2, which support and hinder bone regeneration, respectively. Besides their phagocytic function, these cells also differentiate into osteoclasts and secrete TNF-α and various ILs balancing bone formation and resorption (30, 31).

In this regard, MDSCs, a type of immature myeloid cells, have recently started to attract attention due to their impact on the bone metabolism and their immunosuppressive capacities. First described as a key modulator in tumor microenvironment, the role of MDSCs is becoming undeniably important during disease progression due to their potential to regulate immune balance and crosstalk with the bone system.

## 3 MDSCs are induced in a chronic inflammatory setting

MDSCs were first discovered in a tumor mouse model. Aggregation of these cells around the tumor site lead to suppression of T-cell induced immunity and boosted cancer metastasis (32). While MDSC has become a comprehensive term to describe a specific origin, phenotype, and immunosuppressive capacities, it covers a heterogeneous group of distinct subphenotypes (33). Since several years, interest in MDSC-related immune regulation has been soaring in different disease settings, including chronic inflammatory diseases, infection and obesity (13). Deepening the understanding of the stimulating factors affecting differentiation of MDSCs may offer novel therapeutic targets.

Together with neutrophils and macrophages, MDSCs derive from the myeloid lineage but gain distinguished immunosuppressive functions during differentiation (34–36). Circulating MDSCs have been found in tumor, autoimmune, and septic patients but not or in very limited quantities in healthy individuals (37). In these chronic inflammatory environments, continuous low-grade stimulation of IMCs skews differentiation to increased generation of MDSCs (13). MDSCs generated under these conditions are poorly phagocytic and display potent immune-suppressive potential. Key factors involved in the differentiation of IMCs are granulocyte-macrophage colony stimulating factor (GM-CSF), G-CSF, and M-CSF (38–41), as well as inflammatory cytokines TNF-α, IL-1β, and IL-6 (41–43). These effectors from the microenvironment stimulate and regulate several intracellular pathways involving various key nodes that are crucial for the survival and immunosuppressive function of MDSCs (44–46).

MDSCs are commonly classified as granulocytic (G-MDSC, also known as polymorphonuclear MDSC, PMN-MDSC), monocytic (M-MDSC), and other subgroups such as early-stage MDSC (e-MDSC) and fibrocytic MDSC (F-MDSC) (47, 48). In humans, MDSCs expresses CD11b and CD33–markers related to immunosuppressive functions, while in mice, CD11b, Ly6C and Ly6G were defined as phenotypic markers (34, 49). Additionally, expression of CD84 has been recently identified on MDSCs in tumor settings (36). However, these markers alone cannot sufficiently phenotype all MDSC subpopulations (34). Besides their shared suppressive capabilities against adaptive immunity, their immunosuppressive capability differs in various nuances. In patients with head and neck cancer, PMN-MDSCs displayed the most prominent immunosuppressive features and have been associated with poor clinical outcome (50), while in a tumor mouse model, MDSCs with monocytic features showed heightened suppressive capability and blocked the T cell responses (51, 52).

## 4 Potential interactions of MDSCs with osteoclasts

Osteolysis occurs in several disease including osteoporosis, autoimmune arthritis, bone infection, and bone metastasis, where osteoclasts surpass the speed of regeneration of osteoblasts (7, 53). Related to the destruction of the cancellous bone microstructure, the trabeculae become thinner and more fragile with larger trabecular separation, subsequently manifesting in reduced bone volume (54, 55). MDSCs are osteoclast progenitors that can break the dynamic balance of bone remodeling in disease.

In inflammation, overactivated osteoclastogenesis can be observed, where monocytes and macrophages are functionally calibrated by various cytokines leading to activation of the receptor activator of nuclear factor kappa-B ligand (RANKL) pathway and receptor osteoprotegerin (OPG). T cells bind to RANK, the receptor of RANKL expressed on osteoclast progenitor cells, while OPG competitively binds to RANKL to hinder the stimulating effect of RANK (18). Other inflammatory components including TNF-α, IL-1, and IL-6 also disrupt the bone metabolism by triggering RANKL expression of osteoblasts, cell fusion, multinucleation, and functional activation of osteoclasts (56–58). The inflammatory cytokines stimulate osteoclasts to eliminate defective bone tissue. At the same time, bone regeneration is inhibited by interfering cells supporting the bone metabolism, particularly osteoblasts, osteocytes, and bone marrow mesenchymal stromal cells (BMSCs). Elevated levels of TNF-α, IL-1α, and IL-7 usually found in chronic inflammatory settings lead to osteoblast apoptosis, negatively affecting the osteogenic capacity of osteoblasts and differentiation of BMSCs (7, 59). Additionally, osteoblasts and osteocytes not only sustain the normal bone mineralization process, but also regulate osteoclast differentiation through secreting soluble proteins, inflammatory cytokines, and through direct cell-cell interactions (2, 60).

MDSCs mainly generate where myelopoiesis takes place including the bone marrow, spleen, and other lymphatic organs, but they can be also reprogrammed from mature myeloid cells in the periphery (37). Besides their immune modulatory ability, MDSCs can differentiate into mature and functional osteoclasts (61–65). An *in vitro* experiment using murine Gr1+CD11b+ MDSCs showed that a combination of RANKL and M-CSF can initiate differentiation into osteoclasts. Additionally, in a fluorescent mice model osteoclast generation was increased after MDSC injection, indicating MDSCs as an origin of these bone-resorbing cells (61). Likewise, allogenic transfusion can increase osteoclast differentiation in inflammation (62). Recently, obesity was also suggested to promote expansion of M-MDSCs and subsequent differentiation to osteoclasts (64, 65). MDSC-induced osteolysis is linked to chronic pathological diseases (36, 38). However, MDSCs are a heterogenous group consisting of several subgroups with different immune functions and capacity to differentiate to osteoclasts.

MDSCs and osteoclasts derive from the myeloid lineage, as do monocytes, macrophages, and dendritic cells. Both, MDSCs and osteoclasts share some common intracellular signaling pathways related to differentiation, proliferation, and osteoclastic cell functions. The osteoclastogenic capability of both cell types are repressed after treatment with bisphosphonates, suggesting a shared pathway in MDSCs and osteoclasts (63). Osteoclast differentiation of MDSCs is initiated by activation of the RANKL and NF-κB pathway (62). RANKL also activates the immune regulatory functions of MDSCs and promotes the expansion of M-MDSCs (66). The role of other pathways that have interactions with RANKL/RANK in osteoclast differentiation is of ongoing investigation (67). Additionally, MDSCs and OCs share similar immunosuppressive functions through secretion of the immunosuppressive cytokines IL-10 and transforming growth factor (TGF-β) (3). Both cell types are also capable of inhibiting the T cell mediated immune response. However, they also share immune regulatory features with mature myeloid cells that support the inflammatory environment. They have been shown to be able to sustain a proinflammatory environment under pathological conditions by presentation of antigens, secretion of proinflammatory cytokines, and inducing proliferation of T effector cells (3, 68).

## 5 MDSCs are a link between the immune and skeletal system

**Figure 1:**
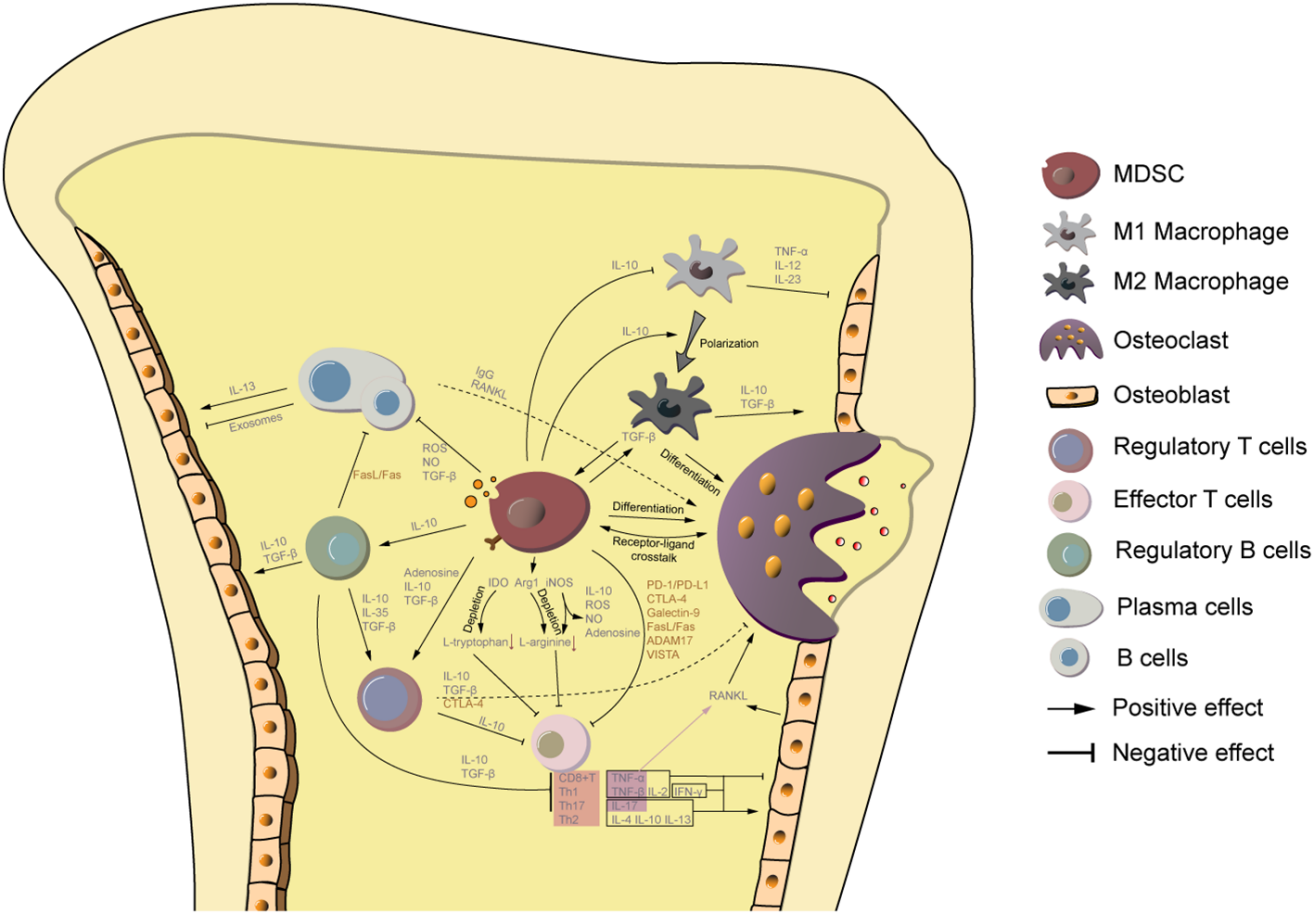
MDSCs are a key link between the bone metabolism and immune system. MDSCs are immature cells of the myeloid lineage that can differentiate to osteoclasts. Additionally, they secret IL-10 to promote macrophage polarization from M1 to M2, of which the latter one is also capable to differentiate to osteoclasts. MDSCs are also involved in the regulation of other counterparts of the immune system. Small molecules from MDSCs, including TGF-β, IL-10, adenosine, and ROS/NO hamper the immune reaction directly or indirectly by supporting proliferation of regulatory T cells, regulatory B cells, M2 macrophages, and inhibiting the activity of effector T cells, B cells, plasma cells, and M1 macrophages. Among them, regulatory T/B cells and M2 macrophage support osteogenic processes. Cytokines from M1 macrophage, CD8+ T cells, and Th1 cells limit osteoblast function, while Th2 and Th17 promote osteogenesis. The effect of plasma cells and B cells on osteoblast activity is controversial and depends on different biological settings. Moreover, immunosuppressive ligands and surface receptors on MDSCs interact with lymphocytes and osteoblasts to regulate their function.

MDSCs also regulate other immune cell types which directly affect the musculoskeletal system. They modulate macrophage polarization from M1 to M2. Anti-inflammatory M2 macrophages stimulate the osteogenic capacity of BMSCs (69, 70). Interaction between MDSCs and regulatory B cells (Bregs) positively impact the bone metabolism (71, 72). Additionally, MDSCs stimulate the proliferation of Tregs that act as key helpers in prolonging osteoblast survival (73). This indicates a complicated interaction triangle among MDSCs, the bone, and components of immune system. **Figure 1** summarizes an overview of the interaction among MDSCs, immune cells and skeletal system.

### 5.1 Soluble factors from MDSCs

A broad range of secreted factors are related to the function of MDSCs, some of which were described immunosuppressive that can prolong the chronicity. However, they also play a versatile role in osteogenesis. TGF-β and IL-10 are two of the most important factors supporting proliferation of Tregs (51, 74), and also play a key role in the generation of osteoblasts (75). Additionally, adenosine which is generated by CD39 and CD73 on the surface of MDSCs can lead to activation of the A2A receptor subsequently increasing production of Tregs (73, 76). Adenosine also has a direct proliferative effect on BMSCs and osteoblasts by activation of the A2B receptor, and therefore contributes to bone regeneration (77, 78). Moreover, other molecules secreted by MDSCSs such as S100A8/A9 and NO have also been shown to positively impact osteoblast differentiation (79, 80).

### 5.2 Immunosuppressive surface markers on MDSCs

Cell-cell contact through immunosuppressive ligands and receptors plays a key in immune dysregulation. Previous studies have suggested a variety of immunoregulatory surface functional molecules to be found on MDSCs (81). These membrane proteins can directly interact with T effector cells, promote the expansion of Tregs and Bregs, and thus regulate systemic immunity in viral infections, autoimmune diseases, and cancer. There are few studies on the direct contact of MDSCs to osteoblasts, but several studies discussed how these surface markers can affect their fate. In particular, the PD-1/PD-L1 axis might have regulatory effect on bone remodeling by limiting osteoclastogenesis (82). Additionally, Galectin-9 is widely expressed in various tissues that were reported to induce osteoblast differentiation (83). CD155, an important receptor mediating cell adhesion, was reported to be expressed on osteoclast precursors and regulate differentiation processes (84). CD276 is membrane-bound but can be also released from the surface as a soluble molecule. Deficiency of CD276 results in lower osteoblastic activity and reduced mineralization (85). Research on ADAM17 demonstrated its role in stimulating osteoclastogenesis by degrading interferon (IFN)-γ (86) and inhibiting osteoblast differentiation through interaction with RUNX2 (87).

### 5.3 MDSC-derived exosome and immune response

Exosomes are a group of lipid bilayer vesicles with nanoscale size (usually 30-100nm), shed by various types of cells during the intercellular communication and regulation. Compared to bone marrow from healthy individuals, exosomes of MDSCs in a tumor environment are excreted in larger numbers and contain more cytokines related to tumor invasion, angiogenesis, and myeloid cell activation or function (88), as well as mRNAs, microRNAs, and other protein molecules involved in immune modulation (89–91). Through proteomic analysis, several typical surface markers on exosomes were found to be representative of their parental MDSCs and beneficial for MDSC migration (92). MDSCs secret exosomes to interfere with their neighborhood in response to changing immune circumstances. CD8+ T cells treated with these small vesicles display a trend towards anergy, while Tregs increase their regulatory activity (88). G-MDSCs were reported to attenuate immune responses of Th1 and Th17 cells and thus reduce the severity of autoimmune arthritis by releasing exosomes (93). Additionally, TGF-β and IL-10 have been found in MDSC-exosomes – two molecules involved in inhibition of autoimmunity and stimulation of osteoblastic growth (94).

## 6 Role of MDSCs in skeletal diseases

MDSCs are activated by inflammation to limit the immune response and to protect against tissue damage. However, in a tumor or chronic bacterial infection environment the immunosuppressive function of MDSCs contribute to disease progression and prolongation. In the skeletal system, MDSCs can not only dampen immune activity, but also cause bone erosion by differentiating to OCs. Despite their importance for bone health, knowledge on their involvement in various different skeletal diseases remains limited.

### 6.1 Ageing and osteoporosis

Osteoporosis is a chronic disease featuring low bone mineral density, pronounced bone loss, bone fragility, and subsequently increased risk for fracture with or without external force. Aging, female gender, genomics, lack of nutrients and other comorbidities are important pathogenic factors impairing bone health and causal to the development of osteoporosis.

Osteoporosis is characterized by gradual degradation of bone tissue with aging. Besides impaired osteoblast function and increasing number of osteoclasts, immune dysfunction has been shown to play a significant role in osteoporosis (6). The aging process of the immune system that is accompanied by progressive immune dysfunction affecting both lymphogenesis and myelogenesis is called “immunosenescence” (95). Specifically, with increasing age there is a gradual decline of T- and B- cells, increased generation of cells from the myeloid lineage, and upregulation of proinflammatory cytokines including IL-6 and TNF-α from senescent cells. The phenomenon of these inflammatory changes within an aging body is called “inflammageing” (96). The resulting chronic proinflammatory environment forms a suitable milieu for proliferation and expansion of MDSCs in bone of the elderly (97–99). Additionally, MDSCs are stimulated towards osteoclast differentiation in inflammageing. Aged individuals show increased MDSC-dependent osteoclast differentiation (99, 100). These changes are driven by increased production of reactive oxygen species (ROS) and nitric oxide (NO). ROS are a set of oxygen-containing molecules aggravating oxidative stress and aging process (101, 102), while NO is synthesized from precursor L-arginine. These molecules damage biologically active molecules, such as DNA, RNA, and enzymes relevant for repairing DNA and cell mitosis (103). In aged individuals, ROS and NO are a potential pathomechanism for enhanced osteoclastogenesis (99, 100). Studies in a murine model of osteoporosis suggest that the resulting bone loss can be alleviated by treatment against these products of oxidative stress (104, 105). Besides being inducers of osteoclastogenesis, ROS and NO function as immune modulators produced by G-MDSCs and M-MDSCs, that suppress T cell generation and function.

Proinflammatory IL-1β, IL-6, and TNF-α, as well as growth factor M-CSF are key regulators in age-related osteoporosis (96, 100). Long-term stimulation by these cytokines leads to increased osteoclastogenesis of MDSCs by upregulation of RANKL – an important regulator of expansion and survival of MDSCs. With increasing age, MDSCs gain more sensitivity to RANKL and are subsequently more stimulated and activated (100). Inhibition of RANKL significantly lowers the proportion of MDSCs vice versa (106). Additionally, chronic NF-κB pathway activation in aged individuals contributes to differentiation of MDSCs (97). The severity of bone loss in osteoporosis is closely related to the activity of the NF-κB pathway (107).

Commonly, bisphosphonates are used to treat age-related osteoporosis. These molecules can dose-dependently abrogate expansion of MDSCs and limit their osteoclastic ability by inhibition of protein prenylation (63), suggesting MDSCs play an essential role in this pathology. Given the impact of MDSCs on the bone metabolism, targeting this cell population is a potential novel therapeutic target against osteoporosis (108).

### 6.2 Autoimmune arthritis and bone destruction

Autoimmune diseases are a range of morbidities characterized by abnormal generation of self-reactive antibodies (4). In contrast to autoinflammatory diseases caused by the innate immune system, adaptive immune cells are responsible for the development of autoimmune diseases. However, both morbidities share inflammation as a common feature. This proinflammatory environment increases osteoclast differentiation and subsequently causes bone erosion as a discernable sign of autoimmune diseases compared to degenerative arthritis.

In autoimmune diseases, MDSCs have been pointed out to be deleterious to bone formation. Charles et al. first described a group of M-MDSC-like myeloid cells with CD11b^-/low^Ly6C^hi^ phenotype with high differentiation potential and myeloid suppressor function in a rheumatoid arthritis (RA) mice model (109). Zhang et al. later identified that co-stimulation of MDSCs with M-CSF and RANKL contributes to bone erosion in a collagen induced arthritis (CIA) model (62). Similar, in another murine autoimmunity model (MFG-E8 knockout mice), bone mass was compromised by enhanced inflammation due to increased osteoclast differentiation of MDSCs (110). In humans, Chen et al. found a strong correlation of M-MDSCs and Th17 cells with osteolysis. Th17 cells can switch to a pro-osteoclastogenic phenotype with high expression of RANKL and reciprocally induce M-MDSCs differentiating into OCs (111). Of note, M-MDSCs were found to secret Arg-1 instead of NO to regulate RANKL expression on Th17 cells (111), which contrasts previous findings that M-MDSCs usually secret NO to modulate the immune responses (13).

Besides their impact on the bone, MDSCs can actively regulate the activity of autoimmune diseases by interacting with T and B effector cells. The immunosuppressive ability of MDSCs has been described in various diseases prone to arthritic lesions, including RA, systemic lupus erythematosus (SLE), and ankylosing spondylitis and the adoptive transfer of allogenic MDSCs has been shown to be a novel treatment approach in affected patients (93, 112). In an autoimmune arthritis model, adoptive transfer of MDSCs skewed the T cell population toward Treg generation, reduced the Th1 and Th17 cell population, and decreased the expression of inflammatory cytokines (113). Similar, transfusion of PD-L1 expressing MDSCs resulted in expansion of regulatory T and B cells and subsequent down-regulation of overactive autoimmunity in a murine SLE model (114).

In contrast to these findings, MDSCs have been reported to prolong or even exaggerate inflammation and thus enhance disease activity. In several reports on the adoptive MDSC transfer in SLE, MDSCs increased disease severity by secreting Arg-1 stimulating Th17 cell differentiation (14, 74). Similar, some reports found higher expression of TNF-α and IL-1β and subsequently increased diseases progression in autoimmune arthritis after MDSC transfer (115, 116). This effect may be caused by selecting MDSCs using Gr-1 and CD11b which can also be found on potentially proinflammatory mature myeloid cells. Another potential mechanism responsible for increased inflammation may be MDSCs potential to differentiate to macrophages or neutrophils depending on the local complex inflammatory environment (117). In addition to an adverse immune response, MDSCs are potential osteoclast precursors when transferred into an autoimmune condition and may deteriorate affected bony structures further.

### 6.3 Orthopedic implant-related infection

Despite increased use of antibiotics and improved aseptic surgical techniques, orthopedic implant-associated infections still remain one of the most challenging complications in orthopedics for patients, physicians, and the health care system alike (118, 119). Chronic inflammation at the bone-implant interface can impact healing and subsequently lead to septic loosening. Once osteolysis sets on, the bone quality decreases over time and the risk for fracture or implant failure significantly increases (118).

In chronic implant-related infection, low virulent bacteria form a layer of biofilm to protect themselves against the immune system and antibiotics (120). Inside the biofilm, bacteria form communities with a reduced metabolic rate, described as a “dormant state” (121, 122). This biofilm gradually elicits the immunosuppressive function of local reactive leukocytes, and therefore prolongs bacteria survival, further complicating successful treatment (16). Additionally, the proinflammatory environment attracts MDSCs to accumulate in the bone niche and attenuate the antibacterial function of polymorphonuclear cells (123).

MDSCs were recently revealed to be involved in the pathogenesis of periprosthetic joint infections. Besides elevated local cell prevalence, their presence in the peripheral blood persists over a long period of time, suggesting a systemic process potentially affecting other organs. However, despite their assumed role in disease progression, knowledge on the impact of MDSCs in implant-associated infections remains severely limited. Their immunosuppressive function has been shown to prolong infection by inhibiting the immune responses mediated by T cells, B cells, and natural killer cells (16, 124, 125). Compared to other myeloid derived cells or lymphocytes, prevalence of MDSCs was particularly high and increased over time in chronic infections (124, 126). Additionally, there has been large numbers of MDSCs observed infiltrating the biofilm, accounting for nearly half of the detectable MDSC population (16). G-MDSCs have been shown to be particularly relevant for heightened bacterial resistance (11). They produce IL-10 leading to increased bacterial persistence (11, 127) and susceptibility to infections (128). After antibody depletion of the G-MDSC population by targeting Ly6G, Ly6C+ monocytes and macrophages expand and regain proinflammatory function essential for clearing bacterial infection (124). Besides G-MDSC, M-MDSC are found around the biofilm albeit in much smaller numbers (16). At the biofilm, M-MDSCs differentiate to anti-inflammatory M2 macrophages that hinder T-cell mediated immunity and thus also contribute to infection persistence (129). Employment of anti-bacterial additions to implants can significantly reduce the number of MDSCs, limit their anti-inflammatory function, and increase efficiency of antibiotics (130, 131). Additionally, successful treatment can positively impact the bone metabolism, as MDSCs differentiate to OCs in infection (132). After surgical addressing of the biofilm, the septic bone destruction recovers significantly (131).

The relationship of the pathogenesis of orthopedic infection and MDSCs is reciprocal. Increased prevalence of MDSCs is linked to heightened risk of infection. Of note, in one *in vivo* human study, the number of G-MDSCs was elevated after aseptic orthopedic surgeries while relative occurrence of total leukocytes and MDSCs remained the same (128). These results suggest during and immediately after surgery risk for bacterial infection may be highest and targeting MDSCs may be a viable prophylactic treatment.

The PD-1/PD-L1 signaling axis has been suggested as a potential target. MDSCs down-regulate T-cell induced pathogen elimination through PD-1/PD-L1 signaling (133, 134). Additionally, *in vivo* experiments suggest a crucial role of PD-1 in differentiation of MDSCs to OCs. PD-1 knockout in osteoporotic mice halved the number of OCs and led to a 2-fold increase in bone volume (82). Inhibition of PD-1 using immune checkpoint inhibitors interrupts OC precursor cell differentiation in areas with bone lesions involving downregulation of CC-chemokine ligand 2/CC-chemokine receptor 2 (CCL2/CCR2) pathway, whereas it exerts no effect on physiological bone structures (135). Conversely, targeting the PD-1/PD-L1 axis may improve clinical outcome, yet can also aggravate inflammation and disrupt the bone metabolism (136). Similar, bisphosphonate can dampen the osteolytic effects of OCs and inhibit MDSC differentiation, however, they have been associated with higher bacterial burden and increased risk for infection (53, 137). Promising novel strategies such as using bisphosphonate as carrier for antibiotics still have to prove effective in a clinical setting (138).

### 6.4 Bone fracture

A traumatic fracture is described as partially or completely disrupted continuity of the bone potentially leading to persisting pain, immobility, and even death due to blood loss (139). However, the bone tissue possesses the potential to fully recover from if treated appropriately. Despite adequate conservative or surgical treatment around 5-10% of affected patients develop mal- or non-union fractures and need additional intervention (140).

Fracture union encompasses consecutive and overlapping phases, from formation of hematoma, soft callus, fibrous tissue to hard callus, and finally remodeled bone (9). The metabolic phases during bone healing interact with the innate and adaptive immune system. The processes involved promote angiogenesis and osteoblast differentiation from BMSCs (9). Dysregulation of the immune response can retard the fracture healing process and is a significant risk factor for mal- or non-union fracture healing. Thus, restoring the physiological immune environment in general and targeting MDSCs in particular is a promising novel therapeutic approach in affected individuals (17, 31).

Currently, there exist conflicting evidence on the role of MDSCs in the bone healing process. Traumatic injury leads to increased cytokine production of IL-1β, IL-6, and G-CSF prompting accumulation of MDSCs (141). Cheng et al. described a long-term dysregulated immune pattern in delayed bone healing (142). By computational analysis, they found a negative correlation of circulating MDSCs and bone healing. MDSCs indirectly suppress the regenerative capability of BMSCs by inhibition of B cell differentiation and elevated IL-10 expression (72). Conversely, MDSCs show a protective effect on injured bone tissue and can even support tissue remodeling (143, 144). After arthroplasty, there is a high concentration of MDSCs that support development of new blood vessel at the polymethyl methacrylate induced periosteal membrane. Local transplantation of MDSCs enhances the formation of these capillaries around the membrane (145). In traumatic fracture healing, significantly elevated number of MDSCs were observed in the transitional area, facilitating the recovery of the bone injury by suppressing local inflammation to stimulate osteoblast differentiation and function (146). However, while MDSCs promote bone regeneration by improving angiogenesis and limiting the inflammatory response, continuous presence of MDSCs pose a risk for infection due to their immunosuppressive capabilities (142).

### 6.5 Bone malignancy and metastasis

Cancer growth depends on both the vigorousness of the tumor itself and a compromised anti-tumor ability of the immune system. MDSCs can facilitate tumor growth through their immunosuppressive capabilities. Research on MDSCs and their involvement in tumor progression has been a main focus and inspires hope for novel therapeutic approaches.

Osteosarcoma (OS) is one of the most prevalent primary bone malignancies in children and teenagers. Both surgical intervention and chemotherapy are employed to enhance quality of life and overall survival. A better understanding of the role MDSCs in supporting growth of OS may open up new treatment options. In the tumor microenvironment, MDSCs, most of them PMN-MDSCs, accumulate and inhibit the T-cell mediated immune responses induced by high expression of IL-18 and CXCL12 (147, 148). Blocking these inducive factors has been shown to sharpen the anti-PD-1 treatment efficacy in mice indicating the importance of the PD-1/PD-L1 axis in MDSCs during the growth of OS (147–149). Activation of the PI3K/Akt pathway was also found to be pivotal in OS tumor growth (148, 149). Additionally, the STAT3 pathway has been related to immunosuppression in this tumor pathology. Inhibition of STAT3 and PI3K/Akt signaling can reverse the suppressive effects on local immunity and reduce tumor size (148–150).

Besides primary bone tumors, the skeletal system is much more commonly affected by metastasis of several types of cancer. In cases of bone metastasis, a variety of growth factors and chemokines produced by the bone and immune regulating cells facilitate the proliferation and expansion of MDSCs (151, 152). At tumor site, malignant cells can precondition the immunosuppressive behavior of BMSCs. These cells subsequently promote the expansion of MDSCs and can attract cancer cells to migrate from the blood into the bone (153). Additionally, MDSCs contribute to epithelial-mesenchymal transition (EMT), thus enhancing mobility, invasion, and resistance to apoptotic stimuli of cancer cells. CXCR2^+^PMN-MDSCs were found to be a major regulator and initiator of EMT through releasing IL-6 during breast cancer progression (154). M-MDSCs can also modulate EMT by secretion of nitric oxide synthase modulate (155). Moreover, MDSCs are involved in the formation of the pre-metastatic niche (PMN). They aggregate at the PMN where they support the construction of the nutritious “soil” for tumor metastases to “plant in” by promoting neovascularization (12, 156) and increasing the activity of neutrophil extracellular traps that can catch circulating tumor cells to colonize (157, 158). Lastly, MDSCs enhance direct differentiation to M2 macrophages (159, 160) and facilitate the differentiation of M1 to tumor-supportive M2 macrophages (161).

The cancer-driven accumulation of MDSCs also has impact on the bone metabolism by differentiating to OCs. This hinders bone regeneration both at the site of osteolytic bone metastases and by dissemination to the bone site *via* blood stream (61). Of note, osteoclast differentiation is MDSC-dependent in bone metastasis, signifying the essential crosstalk between tumor cells and myeloid progenitors in the bone microenvironment (162). Once tumor cells spread to the bone and meet the primed MDSCs they start a continuous stimulate each other reciprocally challenging the bone health. In multiple myeloma, the impact on the bone is even more severe as this malignancy originates from the bone marrow (63). Additionally, the generated OCs enhance tumor immune evasion of multiple myeloma cells from T cell surveillance *via* PD-L1, galectin-9, and CD200 (163, 164). Treatment with immune checkpoint blockers targets this mechanism to revert the MDSC-driven anti-tumor immunosuppression (147).

## 7 Conclusion

The delicate balance of bone resorption and regeneration interacts with and is influenced by the regulatory immune system both physiologically and in disease. In this review, we discuss the impact of MDSCs on the bone metabolism under several pathological conditions, the involved modulatory pathways as well as potential therapeutic targets in MDSCs to improve bone health. MDSCs have a regulatory function on the immune system and can significantly and lastingly impact the process of bone remodeling through differentiation into osteoclasts. In chronic inflammatory conditions, generation of MDSCs is induced. MDSCs have previously been identified in several diseases affecting the bone including tumor, autoimmune diseases, fractures, and infection. They are part of a complex network in which they interact with and regulate other immune cells by releasing soluble proteins, exosomes, and through surface protein-receptor interactions. However, there remains paucity on several of the involved pathways linking MDSCs to osteoclast differentiation and function as well as osteoblast activity and behavior. Emerging evidence suggests a key role of MDSCs in these diseases making them a promising target for novel therapeutic approaches in several diseases.

## Author contributions

Conceptualization, YR. Project administration, MM. Resources, MM. Supervision, AK and MM. Visualization, YR. Writing – original draft, YR, HB, and AK. Writing – review & editing, YR, HB, AK, and MM. All authors contributed to the article and approved the submitted version.
